# Phase-Optimized Multi-Step Phase Acoustic Metasurfaces for Arbitrary Multifocal Beamforming

**DOI:** 10.3390/mi14061176

**Published:** 2023-05-31

**Authors:** Jianxin Zhao, Xiongwei Wei, Chunlong Fei, Yi Li, Zhaoxi Li, Lifei Lou, Yi Quan, Yintang Yang

**Affiliations:** School of Microelectronics, Xidian University, Xi’an 710071, China; jianxinzhao@stu.xidian.edu.cn (J.Z.); 22111213888@stu.xidian.edu.cn (X.W.); 21111212942@stu.xidian.edu.cn (Y.L.); lizhaoxi@xidian.edu.cn (Z.L.); loulifei@mail.xidian.edu.cn (L.L.); quanyi@xidian.edu.cn (Y.Q.)

**Keywords:** acoustic metasurface, multifocal beamforming, Fresnel lens, multi-step phase, acoustic tweezers

## Abstract

Focused ultrasound featuring non-destructive and high sensitivity has attracted widespread attention in biomedical and industrial evaluation. However, most traditional focusing techniques focus on the design and improvement of single-point focusing, neglecting the need to carry more dimensions of multifocal beams. Here we propose an automatic multifocal beamforming method, which is implemented using a four-step phase metasurface. The metasurface composed of four-step phases improves the transmission efficiency of acoustic waves as a matching layer and enhances the focusing efficiency at the target focal position. The change in the number of focused beams does not affect the full width at half maximum (FWHM), revealing the flexibility of the arbitrary multifocal beamforming method. Phase-optimized hybrid lenses reduce the sidelobe amplitude, and excellent agreement is observed between the simulation and experiments for triple-focusing beamforming metasurface lenses. The particle trapping experiment further validates the profile of the triple-focusing beam. The proposed hybrid lens can achieve flexible focusing in three dimensions (3D) and arbitrary multipoint, which may have potential prospects for biomedical imaging, acoustic tweezers, and brain neural modulation.

## 1. Introduction

Focused ultrasound, which has a certain penetration depth and forms a high-energy beam in the focal plane, is commonly utilized in tumor ablation [1,2], non-contact drug delivery [3,4,5,6], neural regulation [7,8], non-destructive evaluation [9,10], and biomedical imaging [11,12,13]. For example, focused ultrasound can penetrate blood vessel walls to accurately locate small areas and ensure that maneuverable particles target movement within the tissue without causing damage to other tissues [5]. Consequently, achieving excellent spatial resolution and high acoustic transmission efficiency is essential for high-energy beams at the target position. Additionally, the proposal of multi-focusing has introduced more degrees of freedom, providing new ideas for high-frequency ultrasound to achieve multifocal beams [14,15]. Compared to single beam focusing, a diversified high-frequency focused acoustic field can achieve multi-target manipulation and obtain more information, which requires simple and novel design methods [16,17].

Commonly used focusing methods include steel ball pressing in flat piezoelectric elements, the phased array with electronic equipment, and the lens fixed to the surface of the acoustic source. The steel ball pressing method [18,19] used in high-frequency single-element transducers with near-perfect acoustic focusing efficiency may damage the piezoelectric sheet and significantly reduce the operating period of the transducer. Phased arrays can theoretically design any acoustic field pattern [20,21,22]. However, high design freedom depends on complex and expensive electronic equipment at the terminal. Acoustic lenses can focus ultrasound without phased arrays or complex terminal electronic equipment, thereby remarkably reducing the size and power consumption of the setup. Lens focusing refers to the design and fabrication of a lens that is placed close to the acoustic source [6,23]. Due to their thickness and high refractive index, traditional half-concave lenses have low transmission efficiency and application range. Therefore, finding a simple and efficient design method for diversified acoustic fields for acoustic wave transmission is urgent and meaningful.

The introduction of metasurfaces has dramatically reduced the size of lenses and broadened the application scenarios, enabling the manipulation of acoustic waves at sub-wavelength thicknesses [24]. Acoustic metasurfaces can modulate transmitted wavefronts to achieve beam steering and focusing [25,26,27]. Accurate phase modulation requires complex topologies, which play a vital role in the design of transmissive acoustic metasurfaces [28,29]. Therefore, to improve the performance of transmitting acoustic metasurfaces, it is necessary to optimize the structure design to minimize impedance mismatching and improve transmission efficiency. The construction of thin Fresnel plate lenses allows acoustic interference to produce a focal point with a higher transmission coefficient than traditional hemispherical concave lenses [30,31]. Fresnel lenses feature planar geometry and relatively easy manufacturing, while high-precision laser processing and 3D-printing technology provide lens-focused fabrication methods [32,33]. Furthermore, transmission efficiency is an essential aspect in the design of acoustic lenses, especially in setup implementation. Based on the multi-step phase Fresnel metasurfaces with a simple structure, efficient transmission and multifocal beam forming can be achieved through practical innovation.

A multivariate acoustic Fresnel lens is designed and fabricated to overcome the abovementioned limitations. A multi-step phase lens is applied to approximate the curvature of the spherical focused field with high acoustic wave propagation efficiency and focusing efficiency by changing the design parameters to obtain different numbers of focused beams without affecting the half wavelength. The acoustic intensity distribution of hybrid lenses is simulated by COMSOL Multiphysics finite element software. A range of three wavelengths with a diameter centered on the focal point covers the entire plane, which can evaluate the amplitude of sidelobes. The phase-optimized hybrid lens reduces the sidelobe amplitude, and excellent consistency is observed between the simulation and experiment of the triple-focusing beam. Furthermore, we conduct the particle trapping experiment to visually display the profile of the triple-focusing beam with optimized metasurface lens.

## 2. Materials and Methods

**Figure 1:**
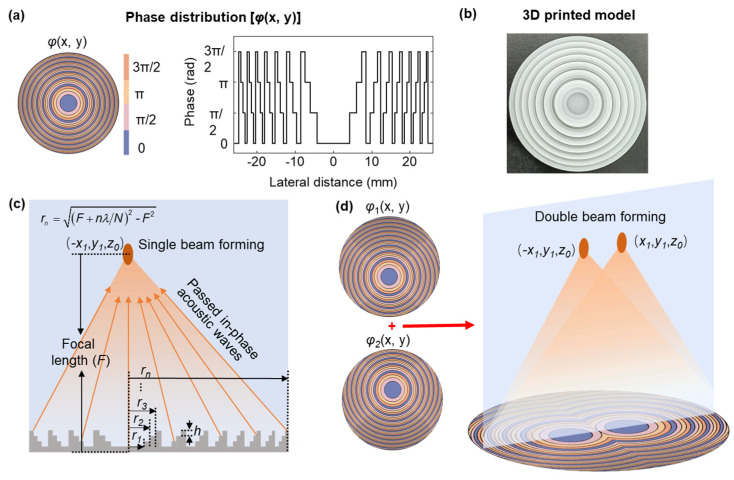
(**a**) Four−step phase Fresnel (FSPF) focusing beam phase distribution and phase φ(x,y) at the initial plane with the beam’s cross-section line of the phase shown on the right side. (**b**) The diagram of the 3D−printed model. Four steps with different heights represent phase difference distributions of 0, *π*/2, *π*, and 3*π*/2, respectively. (**c**,**d**) Cross-sectional diagram of the FSPF lens, indicating how single-point focusing beams and multifocal beams are formed by the FSPF lens.

Figure 1a shows the schematic diagram and phase distribution of the four-step phase Fresnel (FSPF) lens for achieving a single beam. Four steps with different heights represent phase difference distributions of 0, *π*/2, *π*, and 3*π*/2, respectively. The ring radii *r_n_* can be expressed as [34]

(1) $$r_{n}=\sqrt{{\left(F+n\lambda /N\right)}^{2}-F^{2}}$$

where *F* is the focal length from the piezoelectric element, *λ* is wavelength in the propagation medium (water: sound speed: 1490 m/s, density: 1000 kg/m^3^), and *N* is 4, indicating four-step phase distribution. To achieve *π*/2 phase shift, as shown in Figure 1c, the longitudinal step *h* of the lens can be calculated as

(2) $$h=v_{w}v_{p}/\left(Nf\left(v_{p}-v_{w}\right)\right)$$

where *f* is the operating frequency of the ultrasonic transducer, and *v_w_* and *v_p_* are the sound speeds of water and lens, respectively. As shown in Figure 1d, the lens was evenly divided into m (number of focal beams) parts to generate a multifocal acoustic beam. The phase difference $\varphi_{n}\left(z\right)$ of ultrasonic wave between the center and the nth Fresnel zone is given by

(3) $$\varphi_{n}\left(z\right)=k_{w}\left(\sqrt{z^{2}+{\left(r_{n}-x_{1}\right)}^{2}}-z\right)$$

where *k_w_* denotes the wave number of the water, and *x*_1_ denotes the distance between the mapping position of the focus on the *x*-axis and the center. From these considerations, if each part follows $\varphi_{n}\left(z\right)$ = *nπ*/2 and cycles at a period of 2*π*, then the acoustic waves generate constructive interference at the focus.

The *m* fan-shaped FSPF lenses with the same radii are spliced into a complete circular hybrid lens. It is worth noting that the angle α of the sector depends on the number of focal points *m*, i.e., *α* = 2*π*/*m*. Applying the hybrid FSPF lens to a single-element transducer can achieve a multifocal beam forming on the focal plane. In the cylindrical coordinates, the particle velocity of the *n*th Fresnel ring can be expressed as $A=A_{0}\exp\left[j\left(\omega t-\varphi_{n}\right)\right]$, where *ω* is the angular frequency, and *A*_0_ is velocity amplitude. Considering the acoustic wave attenuation $10^{\alpha \cdot h/20}$ in the FSPF lens [35], the acoustic pressure at focus *F*(*x*_1_, *y*_1_, *z*_1_) generated by *dS* is given by

(4) $$dP(x_{1},y_{1},z_{1})=10^{\alpha \cdot t/20}\frac{jk_{w}\rho_{w}v_{w}A_{0}}{2\pi }\frac{e^{j\left(\omega t-k_{p}t-k_{w}l\right)}}{l}dS$$

where *α* and *k_p_* is the attenuation coefficient and wave vector of photosensitive resin, respectively, the *k_w_* and *ρ_w_* are the wave vector and density of water. The *t* is the lens height at different Fresnel rings. The distance *l* from the lens surface *dS* to the focal point *F* can be calculated as $l=\sqrt{{\left(r\cos\theta -x_{1}\right)}^{2}+{\left(r\sin\theta -y_{1}\right)}^{2}+{\left(t-z_{1}\right)}^{2}}$. Therefore, further introducing a fourth-step phase difference in the radial direction can obtain the acoustic pressure of the multifocal acoustic beams as

(5) $$P\left(x,y,z\right)=\sum_{n=1}^{m}\sum_{t=t_{b}}^{t_{b}+3h}\frac{1+\cos\gamma }{2}10^{\alpha \cdot t/20}\frac{jk_{w}\rho_{w}v_{w}A_{0}}{2\pi }\int_{o}^{R}\frac{e^{j\left(\omega t-k_{p}t-k_{w}l\right)}}{l}rdr$$

where *γ* represents the diffraction angle between the focus *F*(*x*_1_, *y*_1_, *z*_1_) and geometric center. Throughout this work, the operating frequency of the ultrasonic transducer is 1 MHz (*λ* = 1.49 mm), and the parameter is *F* = 23 mm (15.5 *λ*). The longitudinal step *h* on the metasurface is calculated as 1 mm for providing *π*/2 phase shift at 1 MHz. The base plate thickness *t_b_* used to stabilize the metasurface is 0.8 mm, which ensures the metasurface’s stability and has excellent transmission efficiency. Depicted in Figure 1b is the four-step phase map which is rendered as a 3D-printing model for the design of an FSPF lens, and the lens material was photosensitive resin (sound speed: 2400 m/s, density: 1600 kg/m^3^).

We designed an ideal mathematical model to verify that the FSPF lens can achieve multifocal beamforming. We calculated the spatial distribution of acoustic field intensity with the commercial finite element analysis software COMSOL Multiphysics 6.0. The geometry is a 3D cylindrical model with a diameter of 50 mm and a height of 50 mm. The physical field for the propagation medium (water) was selected as the pressure acoustics frequency domain to simulate acoustic wave propagation accurately. The ultrasonic transducer was installed at the bottom of the lens, and the piezoelectric sheet was set to an incident radiation pressure of 1 Pa. To simulate the infinite water domain and avoid the influence of non-experimental factors, a perfect matching layer (PML) was selected on the outer side of the model. At last, a sufficiently small mesh (less than *λ*/5, *λ* wavelength at center frequency) was divided, and an ultrasonic wave with a center frequency of 1 MHz was applied to simulate the acoustic field.

**Figure 2:**
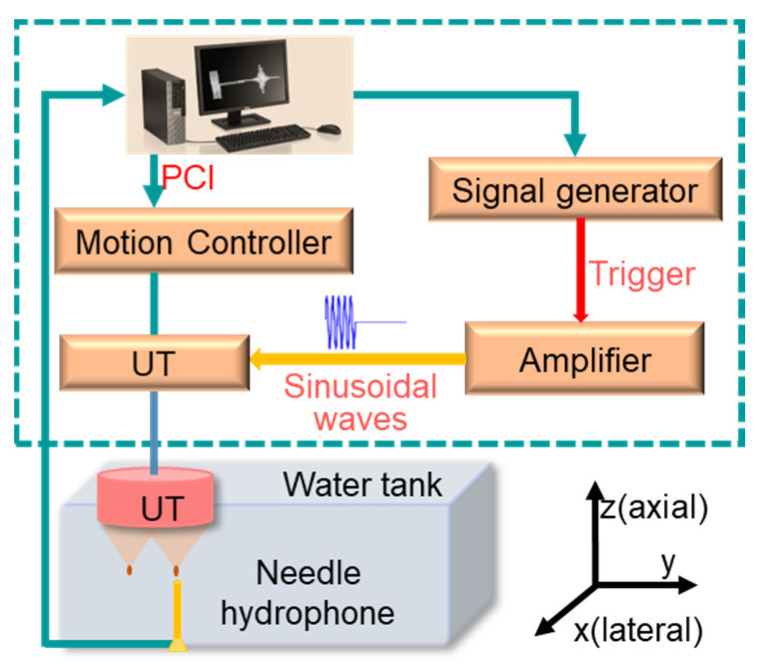
Experimental setup for measurement of acoustic field distribution.

To validate the simulation results, a setup, as shown in Figure 2, was established to measure the spatial distribution of acoustic pressure intensity. Using the ultrasonic gel, a photosensitive resin 3D-printed lens was tightly bonded to the surface of the ultrasonic transducer, and the acoustic waves passing through the lens formed a multifocal beam in water. The ultrasonic transducer was driven by a signal generator (SMB100A, Rohde Schwarz, Columbia, MD, USA) and an amplifier (175A400, ARWORLD, Souderton, PA, USA). A needle hydrophone (NH1000, Precision Acoustics, Dorchester, UK) was fixed at the bottom of the water tank to receive acoustic waves. A 3D-motion system with a step size of 50 μm was controlled by a computer to obtain a 2D acoustic intensity distribution map. The signal was processed in the QT 1140 acquisition card and transmitted to the computer. Finally, offline processing was performed using MATLAB software (R2018a, Math Works, Natick, MA, USA) to form an acoustic intensity map.

## 3. Results

### 3.1. Four-Step Phase Fresnel for Single Beamforming

**Figure 3:**
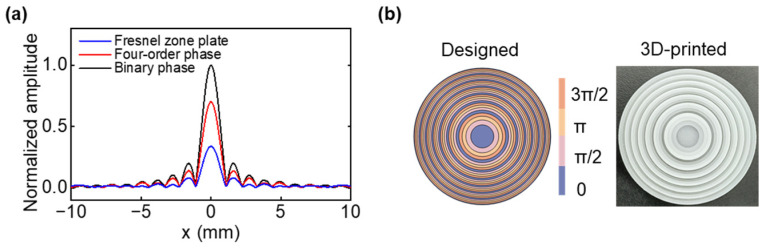
(**a**) Normalized lateral acoustic pressure profiles obtained by Fresnel-zone plate lens, binary-phase Fresnel lens, and FSPF lens. (**b**) Designed and 3D-printed single-point focusing FSPF lens, respectively.

With the goal of performing an investigation of transmission efficiency, different focusing methods, such as the Fresnel-zone plate lens, binary-phase Fresnel lens, and FSPF lens, were selected and studied at a center frequency of 1 MHz. The normalized lateral acoustic intensity profiles obtained by different lenses were calculated, as shown in Figure 3, and were normalized to the maximum amplitude obtained by the FSPF lens. Compared with other lenses, it is evident that the FSPF lens has the highest acoustic intensity value with the highest transmission efficiency at the focal point. Therefore, a four-step phase lens improves the focusing efficiency of acoustic waves after passing through the lens. In this work, a four-step phase lens was designed and fabricated by the 3D-printed method, as shown in Figure 3b. Four different colors represent the distribution of the phase. The diameter of the FSPF lens is 50 mm, with a total thickness of 3.8 mm.

**Figure 4:**
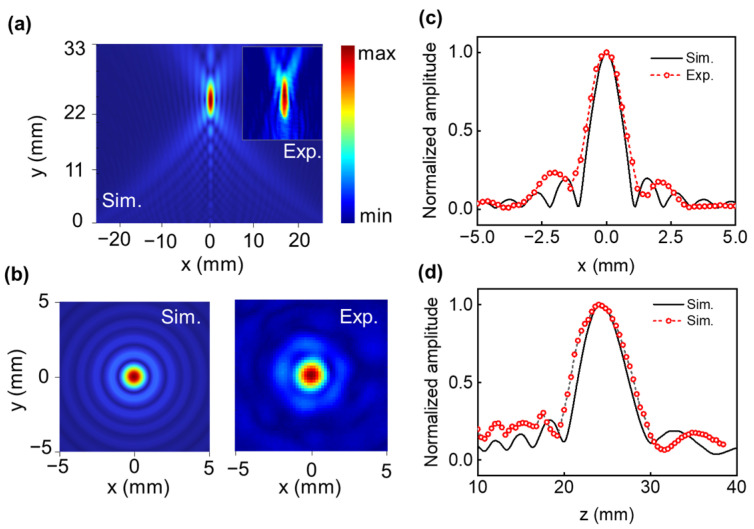
(**a**,**b**) Simulated and experimentally measured acoustic intensity distribution of single-point focusing the beam at x–z and x–y planes, respectively. (**c**,**d**) Simulated and experimentally measured axial and lateral single-point focusing beam profiles across the focal point.

Figure 4a shows numerically simulated and experimentally measured acoustic intensity distribution at the x–z plane, and the ones for the x–y plane are presented in Figure 4b, respectively. As predicted, the maximum acoustic intensity occurs at z = 23 mm, and the focus in the axial direction is 23 mm from the FSPF lens interface. Figure 4c,d present the axial and lateral beam profiles across the focal point. The numerical simulation and experimentally measured results show that the FSPF lens has excellent focusing behavior with an FWHM (full width at half maximum) of 1.51 *λ*. The measured acoustic intensity distributions are in good agreement with the numerically simulated results.

### 3.2. Arbitrary Multifocal Beamforming

**Figure 5:**
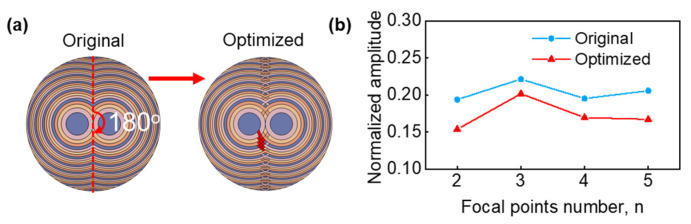
(**a**) Double-focusing beam phase distribution at x = −8 mm with an FSPF lens. (**b**) Normalized original and optimized sidelobe within a range of 3 *λ* from the focus.

FSPF lenses can flexibly adjust their focus in space by changing the design parameters. Figure 1d shows the principle of multifocal beamforming. The *m* fan-shaped FSPF lenses with the same radii are spliced into a complete circular form. The hybrid lens with *m* fan-shaped FSPF lenses was designed and printed for off-axis beam focusing. It is worth noting that the multifocal beamforming of the original hybrid lens introduces sidelobes, which is not expected in biomedical imaging and acoustic tweezers. To address the above problem, we defined the intersecting part of the hybrid lens, as shown in the red parts in Figure 5a, and randomly generated its phase distribution. Statistical analysis of the sidelobe amplitudes within the range of three wavelength radii, with the focal point as the origin, was performed on the simulated acoustic field distribution map to obtain the optimal phase distribution map of the lens. Figure 5 shows the phase distribution of the original and optimized hybrid FSPF lens for double-focal-point focusing. The lens can be divided into sectors with an aperture angle of 180° by a red dotted line. Figure 5b presents the amplitude of the original and optimized sidelobes for multifocal points focusing, indicating a significant decrease in amplitude.

**Figure 6:**
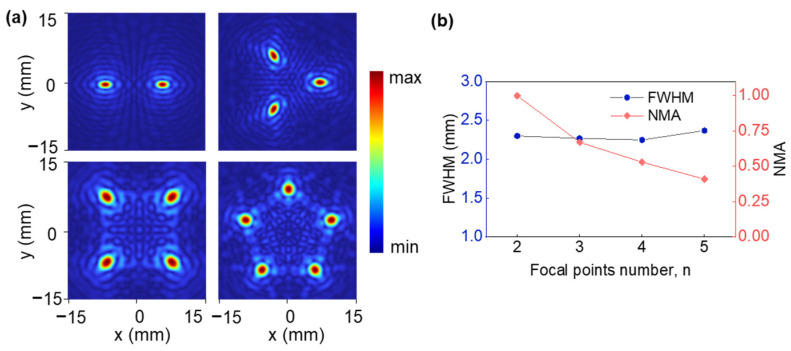
(**a**) Simulated acoustic intensity distribution of the multifocal beam. (**b**) FWHM and NMA at the focus for different focal point numbers.

Figure 6a presents a simulated acoustic intensity distribution with an optimized hybrid FSPF lenses of 2–5 foci at the x–y plane. Multifocal beams are formed with an optimized hybrid FSPF lens from simulation results. Additionally, the FWHM and NMA (normalized maximum amplitude) across the focus are shown in Figure 6b for different focal-point numbers. For the different number of foci, the FWHM remains consistent with single-point beam results (1.51 *λ*), while the NMA of the five-beam focusing lens gradually decreases to half of the amplitude at the focus of the double-beam focusing lens.

**Figure 7:**
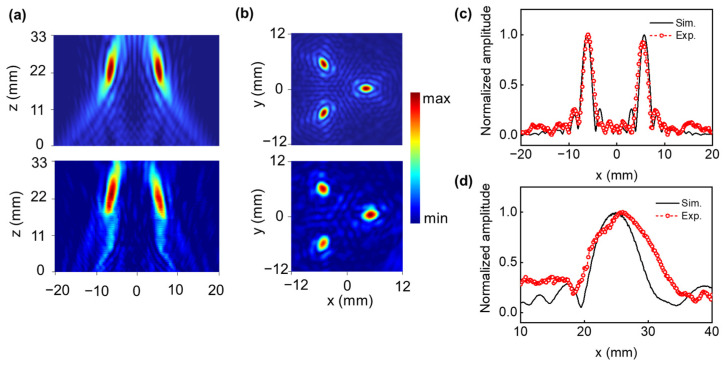
(**a**,**b**) Simulated and experimentally measured acoustic intensity distribution of triple-focusing beam at x–z and x–y planes, respectively. (**c**,**d**) Simulated and experimentally measured axial and lateral profiles across the focal point for triple-focusing beam, respectively.

To further validate the arbitrary multifocal beam-forming behavior of the optimized lens, a hybrid FSPF lens for triple foci was designed and printed. Figure 7a shows the numerically simulated and experimentally measured acoustic intensity distribution at the x–y plane, and the ones for the x–z plane are presented in Figure 7b. As predicted, the maximum acoustic intensity for off-axis beam focusing occurs at x = 8 mm from the lens center, and triple foci were observed. Additionally, double foci were formed in the acoustic intensity distribution map at the x–z plane with x = −8 mm. Figure 7c,d present the axial and lateral beam profiles across the focal point. The numerical simulation and experimentally measured results show that the FSPF lens has excellent focusing behavior with an FWHM of 1.51 *λ*. The FWHM and focal length for arbitrary multifocal beams is consistent with the simulation and the experiment.

### 3.3. Contactless Particle Trapping Experiment

**Figure 8:**
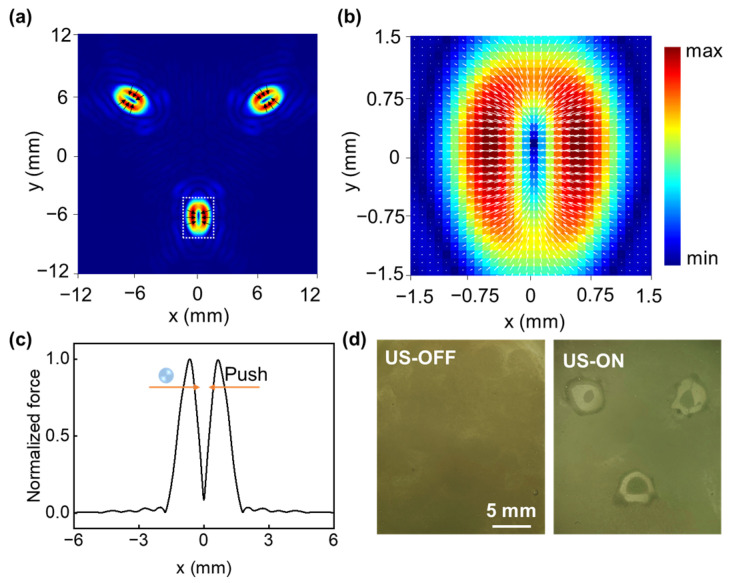
(**a**) Calculated acoustic radiation force field of particles with a diameter of 30 μm. (**b**) Acoustic radiation force for a single focal point in (**a**) circled by a white dashed line. (**c**) Normalized force passing through the center of the x–y plane. (**d**) Profile of the triple focusing beam with optimized metasurface lens.

To display the profile of the triple-focusing beam with optimized metasurface lens, we conducted the particle trapping experiment. Figure 8a shows the calculated acoustic radiation force field of polystyrene particles (sound speed 2400 m/s; density 1050 kg/m^3^) with a diameter of 30 μm, where the color bars and arrow directions represent the amplitude and direction of the force vector in the x–y plane. Figure 8b shows the distribution of the acoustic radiation force for a single focal point in Figure 8a circled by a white dashed line, and the direction of the force points towards the center. We take the center point as the original coordinate point, and the particles away from the center will be pushed to the original coordinate point. The normalized force was shown in Figure 8c, and the radiation force effect acts like acoustic tweezers, causing particles to be trapped in stable potential wells. Polystyrene particles with a density higher than water and a positive acoustic contrast factor will experience a radiation force towards the standing-wave pressure node and display the profile of the triple-focusing beam. As shown in Figure 8d, when the voltage is applied, the radiation force acts on the particles and is tightly squeezed into the center to form three dot profiles.

## 4. Discussion and Conclusions

In conclusion, we present a multifocal beamforming method based on the four-step phase Fresnel lens, and the phase-optimized lens effectively reduces the amplitude of the sidelobe. Firstly, we demonstrated using a planar ultrasonic metasurface composed of four-step phases to achieve automatic beam focusing, which improves the transmission efficiency of acoustic waves as a matching layer and enhances the focusing efficiency at the target focal position. Then the number of focal points and focal length can be adjusted, which reveals the flexibility of achieving arbitrary focusing while maintaining excellent FWHM. We further optimized the phase of hybrid lenses and validated the effectiveness of triple-focusing beamforming metasurface lenses with a measured acoustic intensity distribution map. Furthermore, we conducted the particle trapping experiment to visually display the profile of the triple-focusing beam with an optimized metasurface lens.

The metasurface operating frequency (1 MHz) proposed in this work is a typical frequency for the biomedical ultrasound. The minimum size of the lens is 0.3 mm, and the fabrication of the lens can be achieved using technologies such as 3D printing and fine machining technologies. The principle of multifocal beamforming is revealed, and any other structure with phase control capability is suitable for implementation. Combining artificial intelligence optimization methods can further improve the efficiency of phase optimization and achieve high-resolution complex acoustic fields with acoustic holography. The proposed multifocal beamforming method provides potential prospects for biomedical imaging, drug delivery, and brain neurotherapy.

## Data Availability

Not applicable.
